# Double-Puncture Arthrocentesis in Arthrogenous TMJ Disorders: Bioviscosupplementation vs. Viscosupplementation a Randomized Controlled Trial

**DOI:** 10.3390/jcm14113750

**Published:** 2025-05-27

**Authors:** David Faustino Ângelo, Henrique José Cardoso, David Sanz, Francesco Maffia, Marcella Sarkis, Beatriz Mota, Francisco Salvado

**Affiliations:** 1 Instituto Português da Face, 1500-493 Lisboa, Portugal; david.sanz@ipface.pt (D.S.); francesco.maffia@gmail.com (F.M.); marcella.sarkis@ipface.pt (M.S.); 27168@chln.min-saude.pt (B.M.); 2 Centre for Rapid and Sustainable Product Development, Polytechnic Institute of Leiria, 2430-028 Marinha Grande, Portugal; 3 Faculty of Medicine, Lisboa University, 1649-028 Lisboa, Portugal; fjsalvado2002@yahoo.com; 4 Serviço de Estomatologia, Hospital Egas Moniz—Centro Hospitalar de Lisboa Ocidental, 1349-019 Lisboa, Portugal; 5 Clinica Universitária de Estomatologia, Centro Hospitalar Universitário Lisboa Norte (CHUNL), 1649-028 Lisboa, Portugal; 6 Maxillofacial Surgery Unit, Department of Neurosciences, Reproductive and Odontostomatological Sciences, University of Naples “Federico II”, Via Sergio Pansini 5, 80131 Nápoles, Italy

**Keywords:** hyaluronic acid, platelet-rich plasma, temporomandibular joint, arthrocentesis, osteoarthritis, regenerative medicine, pain management

## Abstract

**Background/Objectives:** Intra-articular injections of hyaluronic acid (HA) or platelet-rich plasma (PRP) have been used in the treatment of temporomandibular joint (TMJ) arthrocentesis to improve lubricative properties and influence regenerative processes. This study aimed to evaluate the potential clinical benefits of complementary bioviscosupplementation with hyaluronic acid (HA) and platelet-rich plasma (PRP) in patients undergoing double-portal TMJ arthrocentesis. **Methods:** A total of forty-six patients (33 females, 13 males; mean age of 45.83 ± 20.62 years) with arthrogenous temporomandibular disorders, identified through clinical and imaging examinations, were randomized into HA+PRP (23 patients) or HA-alone (23 patients) groups. The primary outcome variable was TMJ arthralgia; the secondary outcome was maximum mouth opening (MMO). All the outcome variables were assessed preoperatively (T0) and at several follow-ups (T1—1 month, T2—3 months, T3—6 months, T4—12 months follow-up). **Results:** The HA+PRP group presents lower TMJ arthralgia levels than the HA group at every follow-up moment (*p* < 0.05, r ≈ 0.3). At T3 and T4, the HA+PRP group presented a higher MMO average than the HA group (*p* = 0.03 and *p* = 0.02; r ≈ 0.3). At T4, the HA group’s success rate was lower than the HA+PRP group (65% vs. 96%), and a higher number of postoperative reinterventions were observed (35% vs. 4%). **Conclusions:** In this study, complementary intra-articular bioviscosupplementation (HA+PRP) following double-portal TMJ arthrocentesis was associated with better clinical outcomes regarding TMJ arthralgia reduction, MMO improvement, and reduced risk of future TMJ reintervention.

## 1. Introduction

Temporomandibular disorders (TMDs) are defined as diseases and disorders related to alterations in the structure, function, or physiology of the masticatory system and that may be associated with other systemic and comorbid medical conditions [1]. Recent meta-analytic evidence suggests that TMDs affect approximately 34% of the global population, although prevalence rates vary by region and age group. In Europe, for example, the prevalence is around 29%, with the highest rates observed among adults aged 18–60 years (41%) compared to younger (<18 years, 18%) and older (>60 years, 32%) populations, underscoring its significance as a public health concern [2].

TMDs are frequently characterized by structural changes in articular disc–condyle coordination, including disc displacements (with or without reduction), sometimes associated with inflammatory or degenerative changes in the synovia/cartilage and surrounding tissues, such as osteoarthrosis (OA) [3]. Common symptoms and signs include joint pain, mandibular movement restriction, impaired mastication, and reduced quality of life.

Treatment approaches for TMDs include pharmacological treatment, physical therapy, splint therapy, arthrocentesis with medication injection, arthroscopy, or open surgery. TMJ arthrocentesis is a minimally invasive treatment option that has emerged as a valuable technique to manage intra-articular symptoms [4]. Double-portal arthrocentesis enables effective TMJ lavage through the application of hydraulic pressure, reducing pain levels by washing out pro-inflammatory proteins and increasing mandibular mobility by releasing adhesions in synovial fluid [5]. However, while effective for symptom control, this technique alone lacks TMJ regenerative potential [6].

To address this limitation, intra-articular injections of hyaluronic acid (HA) and platelet-rich plasma (PRP) have been used with arthrocentesis to improve patient outcomes [5,7,8]. HA is a polysaccharide glycosaminoglycan found in cartilage and synovial fluid and is largely responsible for the lubricative properties in humans [9]. Chondrocytes and synoviocytes produce it and play a vital role in elasticity and viscosity, contributing to synovial fluid’s elasticity and shock-absorbing function [10]. In degenerative conditions, such as TMJ OA, a reduction in lubrication was detected due to a decrease in the concentration and molecular weight of HA [10,11]. HA injections have been proven to be effective in reducing TMJ pain [12,13]. However, some studies have shown that viscosupplementation with HA did not have a beneficial effect compared to other treatments, such as corticosteroid injections [14,15]. PRP is an autologous blood product rich in platelets and growth factors, including PDGF, TGF-β, VEGF, and IGF, which stimulates cell proliferation, differentiation, and angiogenesis [16]. These regenerative processes are crucial in modulating inflammation, supporting chondrocyte viability, and restoring extracellular matrix composition [17]. Intra-articular PRP has shown promise in promoting cartilage repair and synovial healing, especially concerning early TMJ osteoarthritic changes [18].

While PRP and HA have been independently studied in TMJ therapy, recent studies have investigated them as a combination therapy (HA+PRP) for their potential synergistic effects. In a study by Wenxing Yu et al., HA and PRP were used in the treatment of knee osteoarthritis, resulting in improved outcomes in terms of pain, function, and stiffness at 52 weeks [19]. While the knee and TMJ differ in regard to their anatomical structure and mechanical function, they share common degenerative features, such as cartilage degradation, synovial inflammation, and reduced intra-articular lubrication. These parallels provide a rationale for examining whether similar biologic therapies may benefit patients with TMJ disorders. Emerging data in the TMJ area suggest that combining HA with PRP may yield enhanced outcomes in terms of pain reduction, joint sounds, and mastication efficiency [20,21].

The combined use of HA and PRP following TMJ arthrocentesis remains underexplored in the literature, and only a few randomized trials have systematically evaluated its long-term effectiveness. Recent systematic reviews have examined the benefits of PRP and PRF (platelet-rich fibrin) in TMJ intra-articular therapy, confirming their safety and efficacy in regard to pain reduction and functional improvement [21,22]. Despite the growing interest in autologous biologics for TMDs, there remains a lack of high-quality randomized controlled trials directly comparing HA+PRP combination therapy to HA alone, following double-portal arthrocentesis. Most existing studies either evaluate these agents in isolation or without a rigorous methodological framework, often lacking control groups, long-term follow-up, or standardized outcome measures. This trial is among the first to address these gaps, employing a randomized, controlled design to evaluate the synergistic effects of HA and PRP on pain, joint function, and the potential for reducing reinterventions. Our study, therefore, contributes important clinical evidence that may inform and optimize minimally invasive treatment protocols for patients with arthrogenous TMDs.

The present clinical study investigates the efficacy of intra-articular infiltration of combined HA+PRP therapy, following double-portal TMJ arthrocentesis. The study’s primary objective is to compare the clinical effectiveness of HA+PRP versus HA alone (viscosupplementation) in reducing the pain intensity in patients with arthrogenous TMDs. The secondary objectives include evaluating improvements in mandibular function (maximum mouth opening) and the need for reintervention during the follow-up period. It is hypothesized that arthrocentesis combined with HA+PRP infiltration will produce superior clinical outcomes across these domains compared to HA alone.

## 2. Materials and Methods

### 2.1. Study Design

A controlled randomized parallel-group clinical trial (clinicaltrials.gov, code NCT06457698) was conducted at the Instituto Português da Face from 1 June 2022 to 1 February 2024. The study adheres to the current legislation, and the principles outlined in the Declaration of Helsinki, with approval given by the Instituto Português da Face ethics committee (PT/IPFace/RCT/0245/14). All the patients signed an informed consent form.

The study was initially designed to include 50 patients (25 in each group). During the study, 4 patients did not conform with the follow-up visits (drop-out rate = 8%). Patients of both genders with a TMD diagnosis and indication for TMJ arthrocentesis were recruited. The inclusion criteria included: (1) age > 18 years; (2) history of conservative treatment for TMDs without significant improvement for at least three months; (3) clinical diagnosis (persisting daily TMJ pain VAS ≥ 4, TMJ sounds, limited mouth opening, painful chewing) and imaging of unilateral or bilateral arthrogenous disorder; (4) radiological findings that most components of the joint were salvageable (assessed by D.F.A) [23]; and (5) magnetic resonance imaging (MRI) and computed tomography (CT) documenting TMJ internal derangement early osteoarthritis alone or combined with other disorders (arthralgia, disc displacement with reduction (DDwR), disc displacement without reduction (DDwoR), disc perforation, bony osteophytes, condylar resorption). The exclusion criteria included: (1) any history of previous TMJ surgical interventions or any facial trauma within the last 4 weeks before the study [24,25]; (2) concomitant surgery on the contralateral TMJ at the time of the study intervention (note, prior contralateral TMJ surgery was not an exclusion criterion if the joint was clinically stable and asymptomatic); (3) severe systemic medical conditions that could interfere with joint healing or the inflammation response, including uncontrolled diabetes mellitus, autoimmune diseases (e.g., rheumatoid arthritis, lupus), bleeding or coagulation disorders, and active malignancy [25,26]; (4) a documented psychiatric illness, including major depressive disorder, bipolar disorder, or schizophrenia, based on the patient’s medical records or self-reported [24]; and (5) pregnancy [27]. No conservative treatment was applied during the follow-up period.

### 2.2. Outcome Evaluation

A diagnosis of TMDs was established according to the Diagnostic Criteria for Temporomandibular Disorders (DC/TMD), using the validated English version [24,28]. All clinical examinations and diagnoses were performed by D.F.A, an experienced clinician trained in the DC/TMD protocol. All the outcomes were assessed preoperatively (T0) and postoperatively (T1—1 month, T2—3 months, T3—6 months, T4—12 months follow-up).

The primary outcome was TMJ arthralgia (VAS, 0–10) [29]. The level of TMJ arthralgia was registered according to the level of pain experienced during palpation of the lateral pole or around the lateral pole or the level of pain on maximum unassisted or assisted mouth opening, right or left lateral movements, or protrusive movements, following the DC/TMD guidelines [24,28]. The patient selected a score for each side, using a 10-point VAS, from 0 representing no pain to 10 representing severe pain. Arthralgia was reported if verified according to (1) a history of pain in the TMJ area and (2) pain modified with jaw movement, function, or parafunction [24,28]. The secondary outcome evaluated was the maximum mouth opening (MMO, mm). MMO was measured, using a certified ruler, between the incisal edge of the maxillary and mandibular central incisors (TheraBite^®^ Jaw ROM Scale). At the end of the study, how many patients relapsed and needed a new intervention was recorded. The success rate of the procedure was graded as successful or a failure, according to Table 1, as described by Eriksson, et al. [30].

### 2.3. Randomization

The subjects were randomly assigned to two groups: HA injection or HA injection + PRP. Codes were prepared and distributed in regard to the two groups for the allocation. The treatment assignment was made by an independent person (nurse), who was not involved in performing any other procedures in the study, such as the preoperative injection or the TMJ arthrocentesis. An envelope containing a code was selected and assigned to the patient. The surgeon (D.F.A) was unblinded because he could distinguish between the color of the two viscosupplements (crystalline for HA alone, amber yellow for HA+PRP).

### 2.4. Treatment Protocol

#### 2.4.1. Double-Puncture TMJ Arthrocentesis Under Local Anesthesia

Local anesthesia was administered, using lidocaine with epinephrine (1:80,000), to block the auriculotemporal nerve. The first puncture involved carefully palpating the lateral rim of the glenoid fossa. A 5 cc syringe was prepared, with a mixture of 3 cc of Ringer lactate and 1.8 cc of lidocaine with epinephrine. A 21 G needle connected to the syringe was gently inserted into the TMJ skin area. Once the needle tip made contact with the posterior slope of the eminence of the upper joint compartment, it was oriented vertically to access the upper compartment, allowing the surgeon to perform a validation step, namely performing a pumping action and observing active and passive pumping, which is crucial for patient safety. For the second puncture, the joint was maintained at maximum distention with continuous inflow through the first portal, while the surgeon felt the insufflation/distention in the anterior joint area. Subsequently, the second portal was established using a 21 G needle, with successful fluid outflow. After completing an effective circuit, joint washing was carried out using intra-articular hydraulic pressure, with at least 150 mL of Ringer lactate solution.

#### 2.4.2. Hyaluronic Acid and Platelet-Rich Plasma Preparation

The protocol was followed according to the guidelines for the use of Endoret^®^ (BTI Biotechnology Institute, Vitoria-Gasteiz, Álava, Spain). Briefly, 32 mL of venous blood per joint was collected and added to four 8ml collection tubes, containing sodium citrate (Endoret^®^, BTI Biotechnology Institute). Then, the blood was centrifugated (Endoret System V Centrifuge^®^, BTI Biotechnology Institute) at 800 rpm for 8 min at room temperature. After centrifugation, 3 layers were perceptible: (1) a layer with platelet-rich plasma and growth factors; (2) a layer with white blood cells; and (3) a layer corresponding to red blood cells. Then, the total plasma volume was calculated, and the plasma corresponding to the first fraction (the low platelet fraction) was carefully aspirated. The second fraction of the first layer corresponding to PRP, above the white blood cell layer, was carefully removed and transferred into new tubes.

#### 2.4.3. Hyaluronic Acid and Platelet-Rich Plasma Administration

Both groups received viscosupplementation (HA group)/bioviscosupplementation (HA+PRP) after completing the articular lavage, using the first portal to access the joint. In the HA+PRP group, ten minutes before mixing with HA, a calcium chloride-rich activator (Endoret^®^, BTI Biotechnology Institute, Vitoria-Gasteiz, Álava, Spain) was added to the PRP. The mixture of activated PRP with HA was then carried out at a ratio of 1 cc of low molecular weight HA (Suplasyn^®^, 20 mg/mL, Inverin, County Galway, Ireland) + 1 cc of PRP and introduced in the joint through the first portal [31,32,33,34,35].

### 2.5. Statistical Analysis

All the statistical procedures were performed using IBM SPSS Statistics 26.0.0 (IBM Co., Armonk, New York, NY, USA) and GraphPad Prism v. 10.0 software (GraphPad Software). The sample size calculation was performed using G*Power v. 3.1.9.7. An effect size of d = 0.80 (large), α = 0.05, and power = 0.95 were assumed, resulting in a required total sample size of 54 participants (27 per group). A final sample of 46 participants (23 per group) was included, yielding an estimated statistical power of 91.4%, which was considered acceptable for the study objectives. Given the non-normal distribution of the variables, as assessed by the Shapiro–Wilk test, non-parametric statistical tests were applied. The Friedman test was used to assess the within-group differences over time. To compare differences between the two groups (HA vs. HA+PRP) at each time point, the Mann–Whitney U test was used. The effect sizes were calculated using Cohen’s *r* (continuous variables) or *Cramér’s V* (categorical variables), computed as small (r ≈ 0.10), medium (r ≈ 0.30), or large (r ≥ 0.50) effect sizes, according to relevant guidelines [36,37]. Statistical significance was set at *p* < 0.05.

## 3. Results

Forty-six patients with a mean age of 45.83 ± 20.62 years old (range 18–88) completed the study, resulting in 83 operated joints. Thirty-three patients were females (71.74%) (Table 1). The preoperative arthrogenous TMD diagnoses were: (1) osteoarthrosis (*n* = 83 joints); (2) arthralgia (*n* = 40 joints); (3) disc displacement with reduction (DDwR) (*n* = 32 joints); (4) disc displacement without reduction (DDwoR) (*n* = 29 joints); (5) osteophytes (*n* = 5 joint); (6) disc perforation (*n* = 3 joint); and (7) condylar resorption (*n* = 3 joints) (Table 2). There were no significant differences in the baseline characteristics between the HA and HA+PRP groups (Table 2). For all the diagnoses and demographic variables, the effect sizes (*Cramér’s V* and Cohen’s r) were negligible, except for the DDwoR, which showed a small effect size (*V* = 0.124).

**Figure 1:**
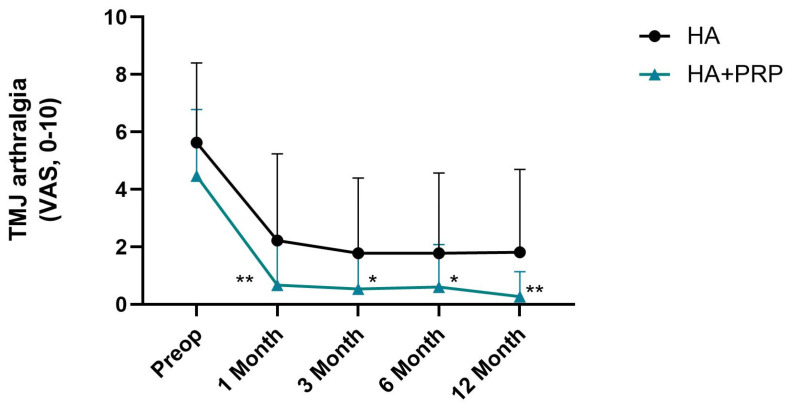
Statistical test results for temporomandibular joint (TMJ) arthralgia comparing the hyaluronic acid (HA) and HA + platelet-rich plasma (PRP) groups. TMJ arthralgia was assessed using the visual analog score (VAS, 0–10). Error bars indicate mean (M) ± standard deviation (SD); the Mann–Whitney U test was used to compare each time frame; * *p* < 0.05, ** *p* < 0.01 when compared to the HA group. HA: hyaluronic acid; PRP: platelet-rich plasma.

**Figure 2:**
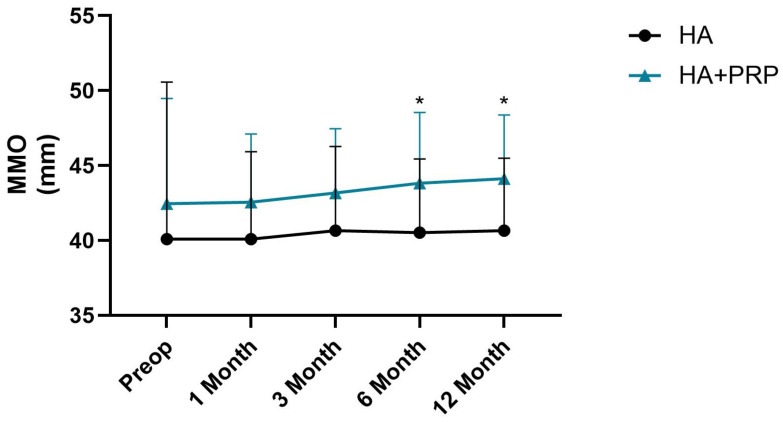
Statistical test results for maximum mouth opening (MMO) comparing the hyaluronic acid (HA) and HA + platelet-rich plasma (PRP) groups. Error bars indicate mean (M) ± standard deviation (SD); the Mann–Whitney U test was used to compare each time frame; * *p* < 0.05, when compared to the HA group. HA: hyaluronic acid; PRP: platelet-rich plasma; MMO: maximum mouth opening.

The mean visual analogue scale (VAS) for TMJ arthralgia before the treatment was 4.46 ± 2.32 in the HA group and 5.63 ± 2.78 in the HA+PRP group. The mean MMO was 40.10 ± 10.48 mm in the HA group and 42.45 ± 7.02 mm in the HA+PRP group. There were no significant differences between the two groups in regard to the preoperative outcomes (Figure 1 and Figure 2).

Significant differences were found within both the HA and HA+PRP groups across the time points (preoperative, 1 month, 3 months, 6 months, and 12 months) in regard to pain (VAS) (χ^2^(4) = 46.51, *p* < 0.001. and χ^2^(4) = 87.25, *p* < 0.001). These results suggest that the pain (VAS) experienced changed significantly over time in both groups, indicating that both interventions had an effect on pain over the follow-up period.

In postoperative phase (T1), a significant reduction in the VAS for TMJ arthralgia was observed in both groups (Figure 1). At T1, the HA+PRP group presented a lower TMJ arthralgia score compared to the HA group (Figure 1, 0.67 ± 1.51 vs. 2.22 ± 3.01; U = 291.50, Z = −2.21, *p* = 0.03). Cohen’s r was 0.29 [0.03, 0.51], indicating a medium effect. At T2 and T4, the statistically significant differences remained similar to T1 between the two groups (Figure 1, U = 296.00, Z = −2.11, *p* = 0.03; U = 287.50, Z = −2.56, *p* = 0.01, respectively). Cohen’s r was 0.280 [0.02,0.50] and 0.339 [0.09,0.55], respectively, indicating a medium effect.

In regard to the MMO outcome, no significant differences between the HA and HA+PRP groups were observed across the time points (χ^2^(4) = 1.40, *p* = 0.84 and χ^2^(4) = 5.16, *p* = 0.27). Comparing the different follow-up moments, no statistical differences were seen at T1 and T2 between the two groups (Figure 2). At T3 and T4, the HA+PRP group presented a significantly higher MMO average compared to the HA group (Figure 2, 43.83 ± 4.72 and 44.13 ± 4.25 vs. 40.52 ± 4.92 and 40.67 ± 4.83, U = 151.00, Z = −2.13; *p* = 0.03 and U = 150.00, Z = −2.16; *p* = 0.03). Cohen’s r was 0.32 [0.03,0.56] for both comparisons, indicating a medium effect.

At the end of the study, the global success rate was 65.22% (n = 15) in the HA group, while it was 95.65% (n = 22) in the HA+PRP group (Table 3, *p* = 0.047). After 12 months (T4), eight patients (34.78%) had an indication for a new intervention. Seven patients were in the HA group, and one patient was in the HA+PRP group (Table 3, *p* = 0.047). The effect size was moderate (*Cramér’s V* = 0.32), favoring the HA+PRP group in both analyses. No harm or unintended effects were observed in either group. Moreover, no adverse events or complications related to TMJ arthrocentesis and the intra-articular injections were observed or reported throughout the study period.

## 4. Discussion

The primary objective of this randomized clinical trial was to compare the clinical efficacy of bioviscosupplementation (HA+PRP) versus viscosupplementation with HA alone, following double-portal TMJ arthrocentesis, in reducing pain and improving mandibular function in patients with arthrogenous TMDs. Our hypothesis was that the combination therapy would produce superior clinical outcomes across all the assessed domains. The results of the study confirmed this hypothesis: patients in the HA+PRP group demonstrated significantly greater reductions in TMJ arthralgia, improved MMO at long-term follow-up, and a lower incidence of postoperative reintervention compared to the HA-only group. Recent developments in regenerative medicine have led to exploring PRP and HA as potential adjunctive therapies in TMJ arthrocentesis. The combination of PRP and HA holds promise due to their complementary mechanisms of action. The term HA “viscosupplementation” indicates the restoration of viscoelastic properties, such as lubrification, elasticity, and anti-inflammatory effects. The term “biosupplementation” refers to the use of biological agents to stimulate tissue repair and regeneration. Clinical studies have demonstrated that PRP is a novel therapeutic agent with several “biosupplementation” therapeutic advantages in treating TMDs, such as joint restoration, cartilage repair, and normalization of endogenous HA synthesis, slowing down OA progression [38]. Marmotti, et al. [39] found that adding HA to PRP could effectively promote chondrocyte proliferation and improve cartilage repair. Similarly, Anitua [40] revealed that PRP may have therapeutic effects in attenuating joint pathology, regarding cartilage healing, chondrogenesis enhancement, and the stimulus of cell proliferation, migration, and differentiation, making it a promising procedure for repairing and replacing damaged articular tissues. HA preparations have relatively short intra-articular half-lives due to rapid clearance and enzymatic degradation by hyaluronidases [38]. However, when combined with PRP, evidence suggests that their therapeutic impact can be extended through synergistic mechanisms. In some studies, the clinical benefits of PRP are maintained for one year after intra-articular injections in patients with knee pain [41]. Previous studies have shown that PRP may enhance HA’s protective effects by improving chondrocyte viability and stimulating extracellular matrix synthesis, thereby contributing to prolonged joint lubrication and anti-inflammatory action [6,38]. Furthermore, this study states that HA lubrification and anti-inflammatory actions might facilitate the distribution and persistence of PRP healing factors within the joint space, concluding that this synergistic interplay between PRP regenerative and anti-inflammatory properties and HA lubricating effects can lead to long-term pain relief and functional improvements [6].

Other studies have also explored the role of platelet-rich fibrin (PRF) in the management of TMJ disorders. Both PRP and PRF reduced pain and improved mandibular mobility in TMJ osteoarthritis [22,42]. Additionally, studies, such as those by Harba, et al. [21,43], confirm the safety and regenerative potential of intra-articular injections of autologous blood products in TMD treatment. These findings support our approach and emphasize the growing interest in biological therapies for TMJ conditions.

The benefits of adjuvant medications, such as HA and PRP, following TMJ arthrocentesis, remain controversial. It is worth mentioning that the literature reveals controversy regarding the superiority of arthrocentesis with supplementation therapy compared to arthrocentesis alone [20]. While increasing evidence supports the use of PRP combined with HA in the treatment of knee osteoarthritis [19,44], their efficacy and mechanisms of action in regard to the TMJ may differ due to joint-specific anatomical, functional, and biochemical environments. Therefore, TMJ-specific evidence is essential before generalizing findings across joints. A recent meta-analysis by Simental-Mendìa et al. revealed that activated PRP was more effective than non-activated PRP in improving pain and function in patients with osteoarthritis (primarily knee OA), but not specifically in regard to TMDs [45]. Platelet activation with calcium chloride (CaCl_2_) induces a less compact fibrin matrix, facilitating a gradual release of growth factors over a seven-day period. This promotes cell migration and wound healing. This effect can be harnessed in autologous gels or scaffolds to ensure the sustained release of growth factors in surgical wound repair. To simplify, activating platelets with calcium chloride stabilizes the release of growth factors, increasing the duration of their effects [46].

Arthrocentesis has long been established as a conservative and minimally invasive approach to the treatment of TMDs. Double-puncture arthrocentesis is a highly safe and effective procedure, facilitating the removal of inflammatory tissue degradants, and its effectiveness in improving TMD outcomes is reported in several articles [5,47]. According to previous studies, the lavage of inflammatory mediators and the lysis of fibrous adhesions between the disc and the glenoid fossa make it a widely accepted and clinically effective treatment in managing TMDs [5,20,48]. Diraçoğlu, et al. [48] compared arthrocentesis with non-surgical treatment modalities, demonstrating that although non-surgical treatment can be beneficial in managing internal derangements, arthrocentesis was proven to have better results regarding the reduction in TMJ pain levels. Gurung, et al. [32] compared arthrocentesis alone with arthrocentesis, followed by HA infiltration. The authors observed that the use of combined therapy was found to be promising and superior to arthrocentesis alone. Al-Moraissi, et al. [49] concluded that minimally invasive procedures deserve to be implemented as efficient first-line treatments or should be considered early on, in other words, as soon as the patient does not show a clear benefit from the initial conservative treatment.

Applying combined supplementation to arthrocentesis in managing TMJ internal derangements has generated increasing interest in recent years. From 2017 to 2023, HA was the most common topic in scientific publications focused on injectables administered into TMJ cavities. However, in the same period, there was a significant upward trend in published primary studies focused on infiltration with PRP preparations in treating TMDs [50]. Cömert Kiliç, et al. [51] conducted a study comparing intra-articular infiltration with PRP with the infiltration of HA, independently, following TMJ arthrocentesis. A group of 49 patients suffering from TMJ OA were divided randomly into two groups, treated with arthrocentesis, followed by intra-articular infiltration with PRP or HA, independently. The outcomes evaluated included VAS pain and MMO. The authors concluded that although both treatment techniques resulted in significant clinical improvements in the VAS and MMO, no statistically significant difference was observed between both groups, suggesting that arthrocentesis plus PRP infiltration was not superior to arthrocentesis plus HA [51].

In our study, we observed statistically significant improvements in TMJ arthralgia (VAS) in both groups; however, the HA+PRP group consistently outperformed the HA-only group in the long term. While the differences were statistically significant, the observed effect sizes (*r* between 0.28–0.33) indicate moderate clinical relevance. These results suggest that the advantage of HA+PRP, though meaningful, may be modest in magnitude at the individual level. In regard to the MMO outcome, no significant differences were seen in the early postoperative stages. Nevertheless, in the latter stages (6 and 12 months post-operation), the HA+PRP group presented higher MMO averages. The effect size for these comparisons was also moderate (*r* = 0.32), supporting a clinically relevant, although not dramatic, benefit. This study corroborates previous studies, as it shows that HA has promising and reliable short-term clinical efficacy in treating TMDs, the postoperative efficacy decreased compared to HA+PRP in combination. In contrast, 6 and 12 months after treatment with the combined therapy, HA+PRP remains significantly effective, with a clear advantage over using HA independently. Additionally, after 12 months (T4), eight patients (34.78%) had an indication for a new intervention, (eight vs. one patient from the HA and HA+PRP group, respectively). This can be explained by PRP’s capacity to promote tissue repair and regeneration, reducing the likelihood of recurrent symptoms and diminishing the need for frequent interventions [51]. At the end of our study, treatment was considered to be successful in 15 patients (65.22%) in the HA group, while in the HA+PRP group, in 22 patients (95.6%). Likewise, the significant reduction in reintervention rates and increased success rate in the HA+PRP group (*Cramér’s V* = 0.321) suggests a meaningful reduction in long-term treatment failure. Our study emphasizes that viscosupplementation with HA alone versus bioviscosupplementation (HA+PRP) has positive effects regarding improvements to functional impairments, MMO, and pain reduction, and a reduced risk of reintervention in patients suffering from intra-articular TMDs. Based on our knowledge, Asadpour, et al. [20] were the first to publish an investigation regarding the clinical efficacy of such treatment and a comparison in terms of arthrocentesis, followed by intra-articular infiltration of PRP, HA, and a combined therapy with HA+PRP, in TMD patients. A total of 30 patients were assessed at six months post-treatment. The established results indicated that although there was an improvement in the clinical outcomes in all the groups, the authors concluded that combined HA+PRP infiltration following arthrocentesis was shown to be more effective than infiltration with HA or PRP independently in the management of TMJ OA. Accordingly, our findings support the hypothesis that combined therapy with HA+PRP infiltration following arthrocentesis can have a significant effect on pain reduction and an increase in the function and MMO in patients with a diagnosis of internal derangements. A study conducted by Hegab, et al. [52] concluded that arthrocentesis with PRP injection exhibits better long-term results, whereas HA injections yield better short-term results. Similarly, Harba, et al. [21] corroborated our results and showed that combined HA+PRP treatment significantly improves the symptoms for patients with TMDs compared to HA alone. Although it was proven that HA was independently effective in alleviating TMJ symptoms in all the follow-up periods, including long-term follow-ups, the combined therapy provided better results regarding the decrease in the mean pain score, improvement in the MMO, and better masticatory efficiency. Taking all the previous findings together, it seems that the regenerative effects associated with PRP enhance the long-term improvements in TMJ health and function. Furthermore, combined HA+PRP has been shown to obtain highly satisfactory long-term clinical outcomes compared to HA independently.

One of the significant limitations of our study is the absence of a group treated with PRP alone following arthrocentesis, as well as the lack of a control group undergoing arthrocentesis alone. However, based on recent studies [20,51,53], which have already demonstrated the efficacy of alone therapy (HA and PRP) compared to arthrocentesis alone, the inclusion of these control groups was deemed non-essential for the primary aim of our trial. Another limitation is the absence of a needle-only (sham or dry-needling) control group, which could have helped isolate the mechanical or neuromodulatory effects of joint injection itself, effects known to occur via local stimulation, similar to dry needling or acupuncture. The decision to exclude a placebo group was based on ethical considerations, as intra-articular penetration without therapeutic intent would involve unnecessary risk. Future studies may consider a three-arm design, including HA, PRP, and placebo/sham groups, to further clarify the independent contributions of the biological and mechanical components in TMJ treatment. Large sample sizes and long-term effects are equally significant in establishing evidence of the long-lasting benefits of combined therapy. Hereupon, incorporating a more extended follow-up period and enrolling a more significant number of patients will be beneficial in evaluating the efficacy of combined therapy in managing and treating intra-articular TMDs. The potential synergy between PRP and HA warrants further investigation through clinical studies that assess their combined effects on pain relief, functional outcomes, and patient satisfaction.

Taken together, these findings align with our results, supporting the view that combination therapy offers both statistical and clinically relevant benefits in pain relief, joint mobility, and a reduction in the need for reinterventions. However, the effect sizes observed in our study were moderate, highlighting the importance of tempered interpretation and the need for further high-quality trials, with larger sample sizes.

## 5. Conclusions

In this study, intra-articular bioviscosupplementation (HA+PRP) injection, following double-puncture TMJ arthrocentesis, had better clinical outcomes regarding TMJ pain reduction, MMO improvement, and functional improvement than HA only. Although statistically significant differences were observed, the corresponding effect sizes were generally moderate, indicating a clinically relevant, but not intense, benefit at the individual level. A reduced risk of future TMJ reintervention was observed in the bioviscosupplementation group. Bioviscosupplementation aims to utilize biological mechanisms to stimulate tissue regeneration. Furthermore, these techniques aim to regenerate damaged tissues, rather than merely alleviating the symptoms, as conventional analgesic pharmacology does. This combined therapy can play an important role in long-term clinical efficacy managing intra-articular TMDs. While the findings are encouraging, they should be interpreted cautiously due to the sample size and the absence of a PRP-only or placebo group. This study contributes to the growing body of evidence supporting the combined use of PRP and HA in managing arthrogenous TMDs and may inform future clinical guidelines. However, larger, multicenter trials with longer follow-up periods and broader outcome assessments are needed to confirm the long-term clinical significance of this approach.

## Figures and Tables

**Table 1:** Criteria for classification of treatment success rate.

Criteria	Description
Successful	No pain or only mild pain level (0–2 on a 0–10 VAS) and MMO ≥ 35 mm
Acceptable	No pain or only mild pain level (VAS ≤ 2 on a 0–10 scale) and MMO ≥ 30 mm and < 35 mm
Failure	Pain constant or moderate (3–10 on a 0–10 VAS) and/or MMO ≤ 30 mm

**Table 2:** Baseline characteristics of the patients. HA: hyaluronic acid; PRP: platelet-rich plasma; *p*: *p*-value. The Mann–Whitney U test was used for continuous variables; Fisher’s exact test was used for categorical variables. The effect sizes are reported as Cohen’s r for non-parametric comparisons and *Cramér’s V* for categorical associations. Confidence intervals (95%) are provided where applicable. A significance level of *p* < 0.05 was considered statistically significant.

	Total (n = 46) (83 Joints)	HA (n = 23) (38 Joints)	HA+PRP (n = 23) (45 Joints)	*p*, *Cramér’s V or Cohen’s r [95% CI] r or V*
Demographic data				
Age M ± SD	45.83 ± 20.62	46.87 ± 23.12	44.78 ± 18.25	0.84, 0.03 [−0.26, 0.32]
Sex (F)	33 (71.74%)	15 (65.22%)	18 (78.26%)	0.51, 0.15 [−0.15, 0.42]
Diagnosis				
Osteoarthrosis	83 (100.00%)	38 (100.00%)	45 (100.00%)	1.00, 0.00 [0.00, 0.00]
Arthralgia	40 (48.19%)	19 (50.00%)	21 (46.67%)	0.83, 0.01 [−0.21, 0.22]
DDwR	32 (38.55%)	15 (39.47%)	17 (37.78%)	0.99, 0.01 [−0.21, 0.22]
DDwoR	29 (34.94%)	16 (42.11%)	13 (28.89%)	0.25, 0.12 [−0.09, 0.33]
Osteophytes	5 (6.02%)	2 (5.26%)	3 (6.67%)	0.99, 0.05 [−0.17, 0.26]
Disc Perforation	3 (3.61%)	2 (5.26%)	1 (2.22%)	0.51, 0.08 [−0.14, 0.29]
Condylar Resorption	3 (3.61%)	2 (5.26%)	1 (2.22%)	0.51, 0.08 [−0.14, 0.29]

**Table 3:** Success rate and need for reintervention in terms of double-puncture TMJ arthrocentesis with hyaluronic acid (HA) compared to HA + platelet-rich plasma (PRP). Fisher’s exact test was used for the categorical variables. Effect size is reported as *Cramér’s V*. Confidence intervals (95%) are provided where applicable. A significance level of *p* < 0.05 was considered statistically significant. HA: hyaluronic acid; PRP: platelet-rich plasma; *p*: *p*-value.

Success Rate				Need for Reintervention	
	Success: Acceptable N (%)	Failure N (%)	*p*, *Cramér’s V*	N (%)	*p*, *Cramér’s V*
HA	15 (65.22%)	8 (34.78%)	0.047, 0.32	8 (34.78%)	0.047, 0.32
HA+PRP	22 (95.65%)	1 (4.35%)	[0.24–0.33]	1 (4.35%)	[0.24–0.33]

## Data Availability

The data presented in this study are available on request from the corresponding author.
